# Revisiting the Intestinal Microbiome and Its Role in Diarrhea and Constipation

**DOI:** 10.3390/microorganisms11092177

**Published:** 2023-08-29

**Authors:** Mihaela Adela Iancu, Monica Profir, Oana Alexandra Roşu, Ruxandra Florentina Ionescu, Sanda Maria Cretoiu, Bogdan Severus Gaspar

**Affiliations:** 1 Department of Family Medicine, “Carol Davila” University of Medicine and Pharmacy, 050474 Bucharest, Romania; adela.iancu@umfcd.ro; 2 Department of Oncology, Elias University Emergency Hospital, 011461 Bucharest, Romania; monica.profir@rez.umfcd.ro (M.P.); oana-alexandra.rosu@rez.umfcd.ro (O.A.R.); 3 Department of Morphological Sciences, Cell and Molecular Biology and Histology, “Carol Davila” University of Medicine and Pharmacy, 050474 Bucharest, Romania; ruxandra.ionescu@umfcd.ro; 4 Department of Cardiology I, “Dr. Carol Davila” Central Military Emergency Hospital, 010825 Bucharest, Romania; 5 Surgery Clinic, Emergency Clinical Hospital, 014461 Bucharest, Romania; bogdan.gaspar@umfcd.ro; 6 Department of Surgery, Carol Davila University of Medicine and Pharmacy, 050474 Bucharest, Romania

**Keywords:** microbiota, microbiome, diarrhea, constipation, probiotic

## Abstract

The gut microbiota represents a community of microorganisms (bacteria, fungi, archaea, viruses, and protozoa) that colonize the gut and are responsible for gut mucosal structural integrity and immune and metabolic homeostasis. The relationship between the gut microbiome and human health has been intensively researched in the past years. It is now widely recognized that gut microbial composition is highly responsible for the general health of the host. Among the diseases that have been linked to an altered gut microbial population are diarrheal illnesses and functional constipation. The capacity of probiotics to modulate the gut microbiome population, strengthen the intestinal barrier, and modulate the immune system together with their antioxidant properties have encouraged the research of probiotic therapy in many gastrointestinal afflictions. Dietary and lifestyle changes and the use of probiotics seem to play an important role in easing constipation and effectively alleviating diarrhea by suppressing the germs involved. This review aims to describe how probiotic bacteria and the use of specific strains could interfere and bring benefits as an associated treatment for diarrhea and constipation.

## 1. Introduction

Gastrointestinal conditions like diarrhea and constipation represent common health problems around the world. Studies show that both diarrhea and constipation are associated with changes that appear in the gut microbial composition [1,2]. Two interchangeable terms are used in the literature: “microbiota” and “microbiome”. Between the two there are subtle differences, because “microbiota” refers to the entire collection of microbial communities found in a specific habitat (e.g., oral cavity, skin, and intestine), while “microbiome” refers to the entire habitat, including the microbial communities, their genomes, metabolites, and the habitat-specific environmental conditions [3,4,5]. The human gut microbiota is a large micro ecosystem containing millions of microorganisms, including bacteria, fungi, and viruses, in perfect equilibrium with the host [6]. Firmicutes, Bacteroidetes, Actinobacteria, Proteobacteria, Fusobacteria, and Verrucomicrobiota are the dominant phyla of the gut microbiota, and 90% of the gut microbial composition is represented by the Firmicutes and Bacteroidetes phyla [7]. In addition to these microbial communities, the intestinal microbiome also includes the totality of genes present in the environment, plus the pathogens [8,9]. The gut microbiota is subjected to diverse influences like host genetics, age, environment, diet and lifestyle, medication usage, etc. [10,11].

Human health is significantly affected by the “core microbiome” alteration [12]. This core microbiome containing specific taxa common to the majority, if not all humans, is very important for host biological functions such as the fermentation of food, resistance to colonization and protection against pathogens, the stimulation of immune response, and metabolite and vitamin production [13]. Microbial balance (eubyosis) is also of major significance in maintaining the integrity of the intestinal barrier [14].

Any disturbance in the composition of the gut microbiota is regarded as dysbiosis and is associated with increased chances of disease occurrence [15]. Dysbiosis can occur due to several exogenous and endogenous factors like excessive/restrictive diet, medication, and immune system and intestinal mucosa integrity [16,17]. Depending on the severity of the dysbiosis, patients can experience a diverse palette of symptoms, from mild effects like cramps, diarrhea, and constipation to more serious chronic conditions [18,19]. Other common symptoms include chronic fatigue; acid reflux and/or heartburn; food intolerance, gas, and bloating; acne, skin rashes, and even psoriasis; inflammation and aching joints; vaginal or rectal infections or itching; attention deficit hyperactivity disorder (ADHD) or issues with concentration; and anxiety or depression [20,21,22]. Dysbiosis has been correlated with a number of conditions like autism, allergic disorders, inflammatory bowel diseases (Crohn’s disease and ulcerative colitis), acute and chronic pancreatitis, and even colorectal cancer [23,24]. Moreover, dysbiosis has been linked to cardiovascular diseases and metabolic disorders such as obesity and type 1 diabetes mellitus [23,25,26,27].

In this review, we will focus on two functional gastrointestinal disorders with temporary or long-term impacts on quality of life: diarrhea and chronic constipation. Diarrhea, especially the infectious one, is considered a major event creator of dysbiosis through increased bowel movements and disruption of the mucosal integrity [28]. Diarrhea is usually treated with antibiotics and probiotics, which leads to a decrease in bacterial diversity. The “washout of the intestine” also contributes to taxonomic scarcity due to the increased amount of water in the fecal matter and reduced transit time [29]. This results in the creation of optimal conditions for the development of facultatively anaerobic germs and a depletion of benefic bacterial metabolites such as short-chain fatty acids [30]. Experimental studies on diarrheal mice have shown that the microbiota diversity and beneficial flora are reduced while the bacterial pathogens are increased [31]. Moreover, the inflammatory factors are increased, and the fecal secretory immunoglobulin A (sIgA) decreases [31,32,33].

A positive outlook is that diarrhea creates a short-lived and reversible dysbiosis. However, the effects on the microbiota should not be overlooked in populations at risk, such as children and the elderly. Probiotics can alleviate diarrhea through several anti-pathogen effects (production of antimicrobial substances, limiting access to nutrients for pathogens, and competitive exclusion) and through general effects like the reduction in gut permeability and stimulating mucosal immune response [34,35,36].

Chronic constipation, including functional constipation and irritable bowel syndrome with constipation, are all characterized by microbiota-mediated intestinal motility disorders. Several clinical studies on probiotics and fecal microbiota transplantation (FMT) suggest that constipation is caused by dysbiosis [37,38,39,40,41]. The most frequently reported results of the studies link constipation with an overgrowth of methanogenic bacteria (*Methaninobrevibacter smithii*) [42]. Methane slows small intestinal transit and appears to do so by augmenting segmental (non-propagating) contractions [43]. Moreover, the degree of methane production seems to be correlated with the degree of constipation [44].

Li et al. found a significant decrease in the abundance of the phylum *Proteobacteria* and butyrate-producing bacteria and a substantial increase in the abundance of *Bacteroides* in women suffering from chronic constipation [45]. Their findings correlate with the study of Zhuang et al., which also describes an abundance of *Desulfovibrionaceae* as important endotoxin producers [46].

Constipation and dysbiosis might also aggravate other coexisting diseases, such as cerebrovascular and cardiovascular conditions, especially in postmenopausal women [47,48]. Vermorken et al. suggested that certain metabolites and cytokines released from mucosal intestinal cells, such as homocysteine, can induce oxidative stress. Oxidative stress, in its turn, may cause intestinal dysmotility and cardiovascular disease [49]. In this direction, Li et al. demonstrated in an experimental study on female Sprague-Dawley rats with induced constipation that sIgA decreases as the content of superoxide dismutase decreases, but the content range of malonaldehyde increases in constipated rats treated with prebiotics [50]. Less frequent bowel movements, as in chronic constipation, are linked to a decline in cognitive function due to a significant decrease in butyrate-producing bacteria [51].

Despite the wide range of medical treatments available for various diseases, the research for complementary therapies with prebiotics, probiotics, and synbiotics has been encouraged by their capacity to regulate the gut microbial composition and improve its functions [52]. This review aims to underline the important role of gut-microbiota-modulating treatment strategies in treating or alleviating symptoms in diarrheal illnesses and constipation, either as a single alternative therapy or as a complementary treatment to current medical strategies.

## 2. Diarrheal Illnesses

Diarrhea represents a common health problem around the world and is associated with a high mortality rate, especially in children under the age of five [53]. Alterations in intestinal fluid water transport the general mechanisms leading to diarrhea apparition [54]. These disorders of water and electrolyte transportation come as a result of damaged intestinal ion transport channel proteins, channels, and physical and chemical barriers [54]. Most cases of acute diarrhea have an infectious etiology [55]. Chronic diarrhea is usually a symptom of celiac disease, chronic pancreatitis, pathological bile absorption, and chronic inflammatory diseases, or a side effect of drug administration (chemotherapy agents, antibiotics, laxatives, proton pump inhibitors, and antihypertensive drugs—beta blockers and angiotensin II receptor blockers), food intake, bowel surgery, and radiotherapy [56,57]. Chronic diarrhea can also be secondary to irritable bowel syndrome (IBS), a chronic functional gastrointestinal disease characterized by changes in bowel habits (diarrhea and/or constipation) associated with abdominal pain [58]. Functional diarrhea represents a particular category of chronic diarrhea, defined according to the Roma IV clinical criteria as diarrheic stools that occur more than 25% of the time, within a time frame from 3 to 6 months before diagnosis, without any structural abnormalities in the intestinal wall [58]. Compared to IBS, the symptoms of abdominal pain and bloating are not predominant in patients with functional diarrhea [58,59]. The intestinal microbiota plays a key role in changes in motility, disruption of the intestinal barrier followed by intestinal malabsorption, generalized inflammation, and modifications in the gut–organ axis [28]. Patients who have Crohn’s disease, an autoimmune pathology of the ileum, one of the inflammatory bowel diseases (IBDs), typically experience diarrhea with mucus or/and blood associated with pain and even sometimes bowel obstructions or fistulas [60]. On the other hand, ulcerative colitis, another IBD, can cause diarrhea in combination with abdominal pain, weight loss, or anemia [61]. The absence of lactase, an enzyme that facilitates the ability to digest lactose in the gut of patients with lactose intolerance, can also be a factor that contributes to watery stools after the ingestion of foods containing lactose [62]. After cholecystectomy, due to the direct excretion of the bile acids in the colon, patients can develop diarrhea, but it can resolve over time without treatment. Chronic infections usually occur in patients with lower socioeconomic status, those who are immunocompromised, or those with recent travel history [60]. A few of the pathogens involved in the process of infectious chronic diarrhea are *Clostridium difficile* (*C. difficile*), *Vibrio cholera*, *Salmonella*, *Shigella*, *Entamoeba histolytica*, *Escherichia coli* (*E. coli*), *Giardia*, and *Cryptosporidium* [63]. Malabsorption disorders are congenital or acquired from surgery defects of the small intestine epithelium that cause the inability of the membrane to transport or absorb nutrients, which leads to diarrheal stools [64]. Chronic diarrhea can clinically be defined as watery, fatty, or inflammatory. This classification can help in the differential diagnosis. Watery diarrhea can be osmotic due to laxative usage, sweetener consumption, or celiac disease. In contrast, secretory and functional diarrhea can be caused by medications (antibiotics, antineoplastics, and others), tumors, bile acid malabsorption, bacterial infections, or IBS. Inflammatory diarrhea is mostly caused by IBDs, neoplasms, or invasive infections with *C. difficile*, viruses, or parasites and can also be a consequence of radiation colitis. Fatty diarrhea appears in cases of severe malabsorption syndromes (tropical sprue, Whipple disease, celiac sprue, SIBO, and gastric bypass) and maldigestion in pancreatic or hepatobiliary disorders [60].

Infectious diarrhea represents a major morbidity and mortality factor, especially in developing countries. Bacterial, viral, or parasitic gut infections cause acute diarrhea and are frequently spread through contaminated water. Most cases of diarrhea are improved in a few days, but severe diarrhea can lead to serious dehydration and become lethal [65]. The main cause of infectious diarrhea is enteric bacterial pathogens, represented by *E. coli*, *Salmonella*, *Campylobacter*, *C. Difficile*, and *Aeromonas* [2,66,67].

*E. coli* is a facultative anaerobic Gram-negative bacteria that is not only a member of the normal gut microbiota but also a well-known gastrointestinal pathogen. Pathogenic *E. coli* species are spread through contaminated water or food and are common in all age groups. Diarrhea caused by pathogenic *E. coli* strains is usually accompanied by fever, which varies from mild to severe. However, mortality is mostly observed in undernourished infants [68]. There are several pathotypes of *E. coli* that cause diarrhea, including enterotoxigenic *E. coli* (ETEC), enteroinvasive *E. coli* (EIEC), enterohemorrhagic *E. coli* (EHEC), and enteroaggregative *E. coli* (EAEC). Each pathotype has different clinical, pathological, and epidemiological characteristics determined by their distinct virulence factors, described in Table 1 [69,70]. Intestinal colonization with *E. coli* is followed by the production of diarrheagenic toxins, such as the Shiga-like toxin produced by EHEC or the heat-labile and the heat-stable enterotoxins produced by ETEC [69]. Enteropathogenic *E. coli* (EPEC) and EAEC are the most frequently involved pathogens in the development of traveler’s diarrhea (TD), a condition with different grades of severity that is defined as three or more unformed stools in a day associated with abdominal pain, vomiting, or fever, which can affect a traveler’s activity [71].

*C. difficile* is a Gram-positive anaerobic spore-forming bacillus present in the intestines of both humans and animals and an important nosocomial pathogen. *C. difficile* is a part of the normal microbiota belonging to the *Firmicutes* phylum, where it is found in a specific range considered normal (2–4% in healthy adults) [72]. Intestinal Clostridiaceae are subdivided into 19 clusters with 120 species, and their growth is suppressed by other anaerobes in a healthy gut [73]. When alterations of the microbiota and the intestinal microenvironment occur, for example, following treatments with antibiotics, proton pump inhibitors, and chemotherapy or when there are associated comorbidities (inflammatory bowel disease, diabetes mellitus, cancer, and pancreatitis) or recent hospitalizations, this creates the premises for *C. difficile* expansion and subsequent infection [74,75]. Therefore, it was shown that for the elderly, where the infection with *C. difficile* is nonresponsive to treatment with metronidazole or vancomycin and is lethal in 15% of cases, the most beneficial intervention is the correction of the microbiota through fecal transplantation [76].

The incidence of *C. difficile*-associated diarrhea (CDAD) has increased in recent years due to antibiotic overprescription [77]. Broad-spectrum antibiotic use, age over 65 years old, past hospitalization, proton pump inhibitor intake, and immunosuppression are the key risk factors for the development of CDAD [78]. The clinical appearance of *C. difficile* infection varies, ranging from asymptomatic or very mild diarrhea to severe pseudomembranous colitis [79]. The pathogenesis of *C. difficile* infection seems to be correlated with the disruption of the normal gut microbial population, primarily from antibiotic treatments like amoxicillin, fluoroquinolones, ampicillin, clindamycin, and cephalosporin that can lead to the development of gut dysbiosis [80]. The commensal flora of a healthy microbiome controls the colonization of pathogens like *C. difficile* through colonization resistance [81]. Following the disruptive effect of antibiotics and antineoplastic or immunosuppressive drugs on the normal gut flora, the intestines are predisposed to pathogen colonization with *C. difficile*, or this effect can lead to an overgrowth of the pre-existing microbial population in the intestinal environment, which can lead to CDAD [80,82]. The pathogen is transmitted via the fecal–oral route and spores, and once ingested can survive gastric acidity and colonize the colon, releasing enterotoxins A and cytotoxin B, which are largely responsible for the clinical severity [83,84]. Studies involving the human microbiota of patients with *C. difficile* infection are correlated with the findings of animal experimental studies [85]. The microbiota of patients with *C. difficile* infection post-antibiotherapy is low in diversity compared to patients who did not develop *C. difficile* infection [86]. Moreover, the microbiota of patients developing recurrent *C. difficile* infection post-antibiotherapy is even lower in diversity compared to those infected who were successfully treated [87]. In addition, the microenvironment containing the microbiota also has a certain impact, although it is less studied. Primary bile acids and certain carbohydrates found in significantly increased amounts favor *C. difficile* growth in comparison with control *C. difficile*-resistant mice [87].

*Salmonella* is a Gram-negative and facultative anaerobic bacterium that causes substantial morbidity and mortality world-wide. *Salmonella* species generally cause gastroenteritis and diarrhea [88]. Based on differences in clinical manifestations, *Salmonella* is divided into *Salmonella typhi* (*S. typhi*) and non-typhoidal *Salmonella*. Non-typhoidal *Salmonella* causes gastroenteritis, while *S. typhi* induces systemic disease, releasing toxins like the Vi antigen and the typhoid toxin and using other virulence factors [89]. Moreover, infections with *S. typhi* occur primarily in developing countries, while non-typhoid *salmonella* has a wide host range [90,91].

*Shigella* is a Gram-negative bacterium and a facultative anaerobic pathogen known to infest the gastrointestinal tract and cause acute shigellosis, with severe bloody and mucous diarrhea. It is transmitted via contaminated food or water or by person-to-person contact [92]. Most cases of *Shigella* infection arise in developing countries and affect the pediatric population under 5 years old [93]. *Shigella* elaborates enterotoxin and serotype toxin 1 in the intestinal lumen, which destroys and invades the intestinal epithelium [94].

*Vibrio cholerae* (*V. cholerae*) is a Gram-negative bacterium with pathogenic and non-pathogenic variants. Pathogenic variants that produce the cholera toxin lead to the acute secretory diarrheal illness called cholera [95]. The effect of the cholera toxins is a massive fluid secretion in the small intestine lumen, which leads to the loss of large amounts of water, sodium, chloride, bicarbonate, and potassium [96]. A metagenomic analysis on cholera patients during and after infection showed that the gut microbiota is highly modified by cholera and then goes through a reproducible pattern of bacterial repopulation [97,98]. This accumulation pattern on bacterial taxa is similar to the maturation of the gut microbiota observed in children [97].

*Fungi* are also part of the gut microbiota. They are, however, only partially understood, because most microbiome studies focus on bacteria. Some fungi might confer health benefits to the host, as is the case for *Saccharomyces boulardii* (*S. boulardii*), a yeast extensively used as a probiotic [99]. However, certain fungi species have been associated with gastrointestinal disorders, including diarrhea, especially in patients with recent antibiotic use [100,101]. *Candida albicans* (*C. albicans*) may cause diarrhea in some cases [102]. Candida species overgrowth has been linked to diarrhea, but the exact mechanism by which the yeast causes diarrhea has not been completely elucidated [103].

Although overlooked, viruses are an important part of the gut microbiome, including ribonucleic acid (RNA) viruses, deoxyribonucleic acid (DNA) viruses, and bacteriophages. Viruses have also been linked to diarrhea, and the most common causative agents are Rotavirus, Caliciviridae (Norovirus and Sapovirus), enteric adenovirus, and Astrovirus [104]. Rotavirus is the most common causative agent for viral diarrhea in infants, and it is associated with high morbidity [105]. Rotavirus is a non-enveloped double-stranded RNA virus that binds to intestinal epithelial cells and through direct entry and Ca^2+^-dependent endocytosis penetrates the enterocyte [106]. Further, it leads to the destruction of absorptive intestinal epithelial cells, causing diarrhea [106].

### Microbiota-Based Therapy of Diarrhea

The gut microbiome has a significant role in maintaining not only intestinal health but general health as well. A healthy gut microbiome can inhibit pathogen intestinal colonization through several mechanisms, including nutrient competition and metabolite production [107]. It has been demonstrated that germ-free mice are unable to eradicate pathogens, whereas, in not-germ-free mice, commensals could outcompete the pathogens [108]. More and more studies are focusing on modulating the gut microbiome to improve human health. The notion of microbiota-based therapy has been discussed in previous studies: it involves dietary interventions, prebiotics, probiotics, synbiotics, antibiotics, and fecal microbiota transplantation.

Diet has a strong impact on gut microbiome health. Specific foods and dietary substances can modulate the intestinal microbial composition and activity and thus confer health to the host [109]. Specific alterations in the gut microbiota following typical food intake represent the subject of recent research in managing the diarrhea-predominant form of IBS (IBS-D) [110]. Evidence shows that the low-**F**ermentable **O**ligosaccharides, **D**isaccharides, **M**onosaccharides, and **P**olyols (FODMAP) diet and the gluten-free diet have positive impacts on IBS-D, while they reduce beneficial bacteria like *Bifidobacteria* and other butyrate-producing microorganisms [111]. FODMAPs are a group of fermentable carbohydrates, which include fructo-oligosaccharides (FOS), galacto-oligosaccharides (GOS), disaccharides, monosaccharides, and polyols [111].

Moreover, it appears that chlorine-containing disinfectants used continuously in freshwater to prevent disease dissemination seem to have negative effects on human health by enhancing the dissemination of antibiotic resistance genes (ARGs) in freshwater and by affecting the intestinal microbiota of zebrafish [112].

Penati et al. suggested that calves receiving waste milk (milk produced by cows treated with antibiotics) might develop diarrhea due to gut microbiome modifications [113]. Exposure to animal-derived foods that may contain antibiotic residues even in low dosages has been linked to gut microbiome modifications that lead to obesity in children. For example, tylosin, an antibiotic growth promoter, can interfere with the gut microbiota by decreasing the Shannon index, thus leading to metabolic imbalances. Evidence suggests that the cooking process of animal-derived food, like frying or roasting, can also enhance the concentration of antibiotics [114]. Although the body can excrete some ingested antibiotics, it has been demonstrated that residues and their metabolites can persist in organisms for a long time at high concentrations [115]. A metabolite of the antibiotic oxytetracycline (OTC), 4-epi-oxytetracycline (4-EOTC), is frequently used in agricultural goods and natural environments as a growth promoter. Residual 4-EOTC effects vary, from increasing the abundance of *Bifidobacteriaceae* and decreasing *Lactobacillaceae* populations in the gut to interfering with amino acid metabolism and sometimes playing a role in inhibiting pathogenic bacteria like *Helicobacteraceae* and *Enterococcaceae* [115].

Despite the wide range of medical treatments available for various diseases, the research for complementary therapies with probiotics has been encouraged by the capacity of probiotics to regulate the gut microbial composition and improve its functions [116].

Probiotics are live microorganisms considered beneficial to the host when administered in the right amount. Species of *Lactobacillus*, *Bifidobacterium*, *Saccharomyces boulardii*, *Clostridium butyricum*, and some species of *Escherichia* are some of the bacteria generally used as probiotics [117]. Researchers have demonstrated that probiotics can reduce the severity of several pathogen infections, such as *Salmonella*, *Citobacter rodentium*, and EHEC [118,119,120]. One study showed that probiotic *E. coli* inhibits the biofilm formation of other *E. coli* strains and of the pathogenic bacteria *Staphylococcus aureus* (*S. aureus*) and *Staphylococcus epidermidis* (*S*. *epidermidis*) [120]. Moreover, studies on murine models have shown that *Bifidobacterium breve* and *Bifidobacterium pseudocantenulatum* DSM20439 can inhibit the expression of the Shiga toxin produced by EHEC [121]. The probiotic intake also appears to reduce the incidence of *C. difficile*-associated diarrhea and antibiotic-induced diarrhea in hospitalized patients [122]. Probiotics containing strains of *Lactobacillus acidophilus* (*L. acidophilus*) and *Lactobacillus casei* (*L. casei*) have offered great results in preventing CDAD, without severe adverse effects reported, and *S. boulardii* also offered great perspectives for the treatment of CDAD, receiving more popularity. Santino et al. described a case of a hospitalized 86-year-old patient who developed fungemia with *S*. *cerevisiae* after receiving treatment with vancomycin and a *S. boulardii* probiotic for CDAD [122,123,124,125,126,127].

When administered in high doses, *Lactobacillus rhamnosus* GG (L. GG) reduces both the duration of diarrhea and the number of stools per day [128]. In addition, L. GG and other probiotics significantly reduce the duration of viral diarrhea, and L. GG appears to reduce Rotavirus shedding [129,130,131]. Similarly, *Lactobacillus reuteri* (*L. reuteri*) DSM 17938 was demonstrated to shorten the duration of infectious diarrhea in hospitalized children as well as in outpatient children [132,133]. Probiotics also appear to be effective in treating dysentery; the duration of both blood in diarrhea and hospitalization time were significantly reduced in patients who received a combination of *Lactobacillus* and *Bifidobacterium* strains and one *Streptococcus* strain [134]. Recent studies suggest that probiotic administration could improve a patient’s compliance to treatment and quality of life and decrease the hospitalization time in the case of *C. difficile* infection [78].

The mechanism by which probiotics alleviate diarrhea is not yet fully understood. However, it is widely accepted that their antidiarrheal properties rely on their capacity to modify the gut microbial composition, strengthen the gut mucosal barrier, and modulate gut mucosal immunity [135,136,137,138]. By increasing mucin expression, some *Lactobacillus* species have been demonstrated to enhance the intestinal barrier and thus exert antimicrobial effects by inhibiting pathogen adherence [136,139]. In one in vitro study, mucin gene expression was increased by exposing intestinal epithelial cells to the probiotic mixture VSL#3 [140]. Moreover, probiotics positively affect tight-junction proteins, limiting the epithelial damage induced by pathogens such as *E. coli* and *Rotavirus* [141,142]. In addition, the exposure of intestinal epithelial cells to *Streptococcus thermophilus* and *L. acidophilus* limited the adhesion and invasion of EIEC [143]. Producing antimicrobial substances, competitive exclusion, competition with cell binding sites, and limiting access to nutrients are also important mechanisms that probiotics use to inhibit pathogen colonization [35,137,144,145]. Probiotics can produce organic acids and metabolites that lower the surrounding pH, creating an unsuitable environment for pathogen multiplication [146,147]. Another important property of probiotics is modulating the intestinal flora composition. A disrupted microbiota with decreased levels of beneficial lactase-producing bacteria such as *Bacillus* spp., *Lactobacillus* spp., *E. coli*, and *Bifidobacterium* spp. can be predisposed to episodes of diarrhea [148]. One advantage of using probiotics in the treatment of *C. difficile* infection is that they have multiple mechanisms of action, such as producing proteases that can directly destroy toxin A and interfere with its receptor sites, down-regulating virulence genes, or directing Quorum Sensing system inhibition [149,150]. Clinical studies have demonstrated that butyrate, a short-chain fatty acid (SCFA), can reduce stool volume in children with *V. cholerae*-induced diarrhea [151,152]. Probiotics can reduce the alterations of the gut microbiota related to antibiotic use and may inhibit the growth of antibiotic-resistant bacteria [153].

Prebiotics can also have a positive impact on patients with diarrhea. Prebiotics are substances that when ingested are utilized by the gut microbial flora and allow specific changes in the composition and activity of the gut microbiome and thus confer health to the host. The main targets of prebiotics are *Lactobacilli* and *Bifidobacteria* [154]. Some studies have reported that prebiotic intake can increase the production of short-chain fatty acids (SCFAs), which are important in maintaining gut barrier integrity [155,156]. Because of its role in promoting normal cell proliferation and differentiation, butyrate is the SCFA considered to have the most beneficial role in intestinal health [154]. Some prebiotics such as galacto-oligosaccharides, fructo-oligosaccharides, inulin, lactulose, and pectin oligosaccharides have been demonstrated to antagonize the adherence of pathogens to epithelial cells, thus inhibiting colonization and promoting gut pathogen elimination [157]. Some studies have shown that prebiotic intake before a trip to a destination with increased traveler’s diarrhea incidence significantly reduced the risk of traveler’s diarrhea [158,159].

Fecal microbiota transplantation (FMT) represents the administration of a solution of fecal matter from a healthy donor into the intestinal tract of a recipient. The purpose of FMT is to change the composition of the gut microbiome and confer a health benefit. FMT has gained popularity over the last few years due to its success in treating gastrointestinal disorders [160]. The benefits of using FMT in patients with diarrhea are based on the idea that the healthy microbial flora introduced via FMT can outcompete pathogens and will restore the composition of a healthy gut microbiome [2]. However, a recent study argues that the benefits of FMT can not only be explained by the restoration of gut bacteria [161]. In a number of studies, FMT has been successfully used in treating refractory *C. difficile*-induced diarrhea [162,163,164]. The existing literature supports the use of FMT and promotes it as a safe and effective treatment for recurrent *C. difficile*-associated diarrhea [165].

## 3. Constipation

Chronic constipation is one of the conditions most frequently encountered by gastroenterologists, and it is associated with a negative impact on quality of life [166]. Between 15% and 20% of adults are affected by chronic constipation, and up to 33% of adults over 60 years old experience it [167]. Functional constipation affects children and adults, with important pathophysiological differences between the two groups [168]. A normal whole-intestinal transit time is 30 to 40 h [169]. Chronic constipation is most frequently idiopathic, but there are cases where it can be secondary to ongoing medication or diseases [170]. Diet, intestinal motility and absorption, anorectal motor and sensory function, behavioral factors, and psychological issues are all part of the pathophysiology of functional constipation [170]. Functional constipation is categorized into constipation with normal transit, slow-transit constipation. and rectal evacuation disorders [168]. Treating chronic constipation can be challenging, and current treatment strategies include dietary changes, treatment of depression, and the ingestion of bulking agents such as psyllium or methylcellulose, stimulating laxative medication, lactulose, and sorbitol [171]. Treatment options for chronic constipation may follow the recent American Gastroenterological Association/ American College of Gastroenterology (AGA/ACG) clinical practice guideline [172].

The enteric nervous system (ENS), the central nervous system (CNS), the immune system, and the intestinal luminal environment are the inter-related factors that control gastrointestinal motility [173]. Constipation symptoms might appear if perturbations occur in any of these systems [173]. The existence of a bidirectional “microbiota-gut-brain axis” [174] and its important role in regulating intestinal motility is supported by growing evidence [173,175]. Some studies have demonstrated that probiotics can positively affect gut motility by modulating the ENS or the CNS [176,177]. Some studies indicate that probiotics can increase gut motility by modifying the microbiota composition and microbial fermentation, which triggers the release of metabolites such as short-chain fatty acids, peptides, and lactic acid that interact with the ENS [173,178,179].

The gut microbial population plays a key role in intestinal motility, and dysbiosis has been correlated with chronic constipation [1]. Studies that have analyzed the microbiota in constipation and constipation-predominant irritable bowel syndrome have shown that there is a decrease in *Bacteroides*, *Bifidobacterium*, and *Lactobacillus* spp. Compared to control groups and an increase in potentially pathogenic bacteria such as *Pseudomonas aeruginosa* and *Campylobacter jejuni* [173,180,181,182,183]. A cross-sectional pilot study using 16S rRNA gene pyrosequencing reported that the microbiota of constipated patients presents a decreased concentration of *Prevotella* and increased concentrations of the *Firmicutes* genera [184]. Parthasarathy et al. also demonstrated the correlation between a more rapid transit time and increased concentrations of *Actinobacteria*, *Bacteroides*, and *Lactococcus* [182].

According to Barbara et al., the intestinal microbiota influences gut motility by releasing bacterial fermentation end-products via intestinal neuroendocrine factors and through mediators released by the gut immune response [185]. SCFAs, products of bacterial anaerobic metabolism, have been demonstrated to stimulate ileal propulsive contractions and appear to be able to directly stimulate ileal and colonic smooth-muscle contractility [183]. Moreover, SCFAs, especially butyrate, exhibit pro-absorptive NaCl and anti-secretory effects toward Cl^−^ secretion [186].

### Microbiota-Based Therapy of Constipation

Dietary fiber has been intensively recommended for treating chronic constipation due to its multiple benefits to intestinal microbiome health. Dietary fibers are known to increase the volume of the stool and soften it, as well as decrease transit time [187]. Dietary fiber acts as a substrate for microbial intestinal fermentation, stimulates the growth of beneficial bacteria, and promotes the excretion of fermentation end-products such as SCFAs, which adversely affect human health when built up [188]. Moreover, it was shown that dietary fiber can stimulate the growth of beneficial bacteria while suppressing pathogenic bacteria [189].

The low-FODMAP diet shows good results in patients with constipation-predominant IBS (IBS-C). However, it also decreases fiber intake, thus leading to aggravating constipation in some cases of IBS-C [112]. Although there is still insufficient evidence to demonstrate the beneficial effect of nutritional approaches on gut microbiota manipulation for the overall improvement of different chronic digestive diseases, diet modifications seem helpful for symptom alleviation [190,191,192].

Prebiotics are non-digestible carbohydrates that promote the health of the host by stimulating the growth of some commensal gut bacteria, such as *Lactobacilli* and *Bifidobacteria* [193]. Prebiotics like inulin, fructo-oligosaccharides, and galacto-oligosaccharides are metabolized in the intestinal lumen and transformed into lactic acid and short-chain carboxylic acid [183]. Studies on mice have demonstrated that prebiotic oligosaccharides stimulate gut peristalsis, thus alleviating constipation symptoms [194]. In the clinical setting, lactulose relieves constipation and increases fecal *Bifidobacteria* counts [195]. In the clinical setting, no significant differences were observed in the relief of constipation when compared to placebo [195,196].

Probiotics are extensively used as alternative treatment options in patients suffering from constipation due to their beneficial effects [197]. Probiotics may benefit patients suffering from chronic constipation by modifying the intestinal luminal environment and changing the composition of altered gut microbiota [198,199]. The consumption of *B. lactis* containing fermented milk increased stool frequency and improved stool consistency and defecation conditions in a population of Chinese women suffering from constipation [200]. In addition, one systematic review demonstrated that *B. lactis* reduced whole-gut transit time, increased stool frequency, and improved stool consistency in patients with functional constipation [173]. One of the most recent trials evaluated the clinical efficacy of multiple strains of probiotics in treating chronic constipation in elderly patients using a multi-probiotic mixture containing *Bifidobacterium animalis* subsp. *lactis* BCL1, *L. acidophilus* LA3, and *L. casei* BGP93 [201]. After 71 days, the cumulative stool number was significantly higher in the probiotic group compared to placebo [201]. Recently, a meta-analysis by Zhang et al. identified 15 randomized controlled trials (RCTs) that investigated the efficacy of probiotic administration in constipation. Gut transit time (GTT), stool frequency, consistency, and bloating were analyzed. The meta-analysis demonstrated that probiotics such as *Bifidobacterium*, *Lactobacillus*, and *Streptococcus* ameliorate functional constipation by increasing stool frequency and decreasing gut transit time and stool consistency. However, symptoms of bloating were not significantly reduced [202]. Compared to single-species probiotics, multispecies probiotics were found to significantly improve symptoms of constipation [201]. The superior results obtained with multispecies probiotic administration may be explained by synergistic interactions between probiotic strains [201,203]. One systematic review of nine RCTs that investigated the clinical efficacy of probiotics in treating constipation in elderly people reported that probiotic therapy significantly improved constipation compared to placebo groups, with *B*. *longum* being the most frequently tested probiotic among the analyzed trials [204].

Synbiotics represent a mixture of probiotics and prebiotics. One randomized controlled study evaluated the efficacy of a synbiotic combination containing *Bifidobacterium longum* (*B. longum*) NCIMB 30182, *Bifidobacterium breve* (*B. breve*) NCIMB 30180, *Lactobacillus casei* (*L*. *casei*) NCIMB1 30185, *Lactobacillus rhamnosus* (*L*. *rhamnosus*) NCIMB 30188, *L. acidophilus* NCIMB 30184, *Lactobacillus bulgaricus* (*L. bulgaricus*) NCIMB 30186, *Streptococcus thermophilus* (*S. thermophilus*) NCIMB 30189, and fructo-oligosaccharides in ameliorating constipation in a population of 66 men with chronic idiopathic constipation [205]. A second RCT used a synbiotic combination of *B. lactis* HN019, *Lactobacillus paracasei* (*L. paracasei*) Lpc-37, *L. rhamnosus* HN001, and *L. acidophilus* (NCFM), fructo-oligosaccharides in a population of women suffering from chronic idiopathic constipation [206]. In addition, studies have demonstrated the efficiency of synbiotics in ameliorating constipation in children with chronic constipation [207,208].

The agents mentioned above are generally well tolerated, and their administration is considered safe. Probiotics, prebiotics, and synbiotics may represent an effective treatment option for patients suffering from chronic constipation. However, further studies are needed to evaluate specific species strains and the optimal treatment dosages and durations.

In patients with chronic constipation, Zhang et al. demonstrated that FMT in combination with soluble dietary fiber had both short-term and long-term efficacy in treating slow-transit constipation [209]. In an early study by Borody et al., FMT demonstrated significant improvement in defecation frequency and symptoms like abdominal pain, early satiety, and nausea [210]. A significant increase in defecation frequency and stool consistency was also demonstrated by Ge et al. in a case series that investigated FMT in six patients with slow-transit constipation [211]. In one RCT, 60 adult patients with slow-transit-time constipation were randomized to receive either FMT or conventional treatment. The FMT group had significantly improved constipation symptoms, demonstrating that FMT effectively treated constipation [40].

FMT was proven effective in treating both refractory diarrhea and refractory constipation. However, this procedure is associated with high risks, and the Food and Drug Administration has issued a warning following the death of one patient after FMT and after one patient developed an infection [212]. Moreover, immunocompromised patients are at risk of developing bloodstream infections if undergoing FMT [213]. Due to the dangers associated with FMT, the challenge in identifying donors, and the complexity of the procedure, only selected cases of patients who are refractory to conventional treatment options should undergo this procedure.

## 4. Comparison of Intestinal Dysbiosis and Therapeutic Approaches between Diarrhea and Constipation

Constipation is the opposite of diarrhea. Chronic constipation stools are hard, lumpy, or even dry and pass fewer than three times a week due to a long transit time, while in diarrhea, stools are watery and loose and pass more than three times a day due to a fast transit time. Although diarrhea and constipation are two opposed manifestations, both are characterized by dysbiosis and the decrease in the number of beneficial bacterial populations, creating conditions for developing potentially pathogenic flora. A study performed by Vandeputte et al. regarding colon microbiota composition and stool consistency showed that fecal microbial richness and community composition are strongly correlated with stool consistency and transit time [214]. Moreover, the gut microbial community structure stratified as three main enterotypes was found to influence the texture of the stool: the *Prevotella* enterotype is more abundant in individuals with loose stools, while the *Bacteroides* enterotype completely dominates firmer samples [214].

In our opinion, any invasive pathogenic bacteria, viruses, or fungi, for example, in the case of infectious diarrhea, might have different effects if the infection occurs in a healthy microbiota or a dysbiotic flora. Our observation is based on the fact that a healthy microbiota is responsible for mucosal immune homeostasis and the production of essential metabolites and several bacteriocins that prevent pathogenic invaders. In support of this theory, we rely on the study of Ward et al. referring to the health of the intestinal and oral microbiomes as predictors of COVID-19 severity and lethality [215]. They found that the presence of intestinal *Enterococcus faecalis* (a pathobiont) is a great predictor of COVID-19 severity, and it was also linked to the activation of an inflammatory immune response involved in the evolution of comorbidities such type 2 diabetes and hyperlipidemia [215]. Eubyosis influences not only the digestive tube but also distant organs such as the brain, liver, pancreas, and lungs, accentuating the negative evolution of the host’s coexisting metabolic problems or chronic diseases [216]. On the other hand, acute infectious diarrhea leads to the worsening of a pre-existing dysbiosis, and if this happens to children, the development and the maturation of the mucosal immune system and the integrity of the intestinal barrier might be affected in the long term, making the children more likely to develop an autoimmune disease due to a leaky gut [217].

Both infectious diarrhea and chronic constipation can be treated with antibiotics. While in infectious diarrhea the antibiotic selection depends on the type of pathogen and is not addressed to reduce the number of potential pathogens found in the dysbiotic flora, in chronic constipation the antibiotics are dedicated to the destruction of methanogenic bacteria in constipation-predominant irritable bowel syndrome [218]. The study by Pimentel et al. conducted in 2014 is in correlation with the previous research by Low et al. conducted in 2010, and states that the rifaximin and neomycin combination is more effective in reducing methane production and significantly lowers constipation severity [219].

In both affections, diarrhea and constipation, microbiota-based therapy has been discussed above. The main common aim is to improve dysbiosis in the long term. In our opinion, this is possible in less severe cases through the administration of pre/probiotics and/or synbiotics that must be sustained by important dietary changes to provide the necessary substrate for each species and family which were externally administered. Though the three main enterotypes were shown to remain stable throughout short- or long-term dietary interventions, preserving the core microbial profile and increasing the number of beneficial bacteria might influence the overall improvement of intestinal health by re-establishing the integrity of the intestinal barrier and the immune response homeostasis [220,221,222]. The modulation of the intestinal microbiota during the recovery phase after diarrhea and in the long term can improve the recurrence and the outcome in future episodes [62,223]. Regarding functional constipation, a study by Arslan et al. hypothesized that “an individualized diet based on microbiome analysis may improve symptoms” [224]. Their results showed that an artificial intelligence (AI)-assisted customized diet based on individual microbiome analyses was better than conventional therapy for constipation [224]. The restoration of the microbiota to a homeostatic state is useful in both diarrhea and constipation, and FMT seems to be an effective therapy in specific cases. FMT is recommended especially in multiple recurrences of *C. difficile* infection, which usually appears in 20–25% of patients, and the effectiveness seems to be equivalent if not superior to antibiotic treatment, according to clinical trials [225,226,227].

In constipation, the re-establishment of the intestinal microbiota was demonstrated to improve intestinal motility [228]. It was shown that FMT was 30% more effective than classical treatments for slow-transit constipation in a randomized, single-blind, placebo-controlled clinical trial performed by Tian et al. [40].

One can perceive FMT as an ultimate solution for cases when antibiotic therapy, dietary changes, and the administration of prebiotics, probiotics, and synbiotics bring no results. There are many controversies regarding this medical procedure. From safety issues to the delivery method of FMT, clinicians should be updated with the regulations in their countries and with the chemical and biological components of FMT (for details, see review [229]).

## 5. Conclusions

The results from clinical studies on probiotics and on FMT suggest that both diarrhea and constipation are caused/affected/aggravated by dysbiosis of the gut microbiome. A healthy microbiome prevents pathogen colonization and thus protects the host from severe forms of diarrhea. Existing studies suggest that preventing and treating diarrheal illness by modulating the intestinal microbiome through alternative treatment strategies like probiotics or FMT is efficient. Given the complexity of constipation, studies should focus on finding causative bacteria and on developing probiotic treatments that can cure constipation in the future. Future studies should focus on developing standardized protocols for specific probiotics. In addition, we would like to emphasize the need for rigorous donor screening in the case of FMT procedures.

## Figures and Tables

**Table 1:** *E. coli* pathotype characteristics.

Pathotype	Diarrhea Type	Clinical Presentation
EPEC	Infant diarrhea	persistent watery diarrhea
ETEC	Traveler’s diarrhea Infant diarrhea	watery, non-bloody stools
EIEC	Dysentery	diarrhea with blood and mucus
EHEC	Hemorrhagic colitis Hemolytic uremic disease	hemorrhagic colitis or watery diarrhea without blood
EAEC	Traveler’s diarrhea Infant diarrhea	persistent watery diarrhea

EPEC, enteropathogenic *E. coli*, ETEC, enterotoxigenic *E. coli*, EIEC enteroinvasive *E. coli*, EHEC, enterohemorrhagic *E. coli*, EAEC, enteroaggregative *E. coli*.

## Data Availability

Not applicable.

## Abbreviations

AI	artificial intelligence
ADHD	attention deficit hyperactivity disorder
AGA/ACG	American Gastroenterological Association/American College of Gastroenterology
ARGs	antibiotic resistance genes
*B. breve*	*Bifidobacterium breve*
*B. longum*	*Bifidobacterium longum*
*B. lactis*	*Bifidobacterium lactis*
*C. albicans*	*Candida albicans*
*C. difficile*	*Clostridium difficile*
CDAD	*C. difficile* induced diarrhea
CNS	central nervous system
COVID-19	Coronavirus disease 2019
DNA	deoxyribonucleic acid
*E. coli*	*Escherichia coli*
ETEC	enterotoxigenic *E. coli*
EIEC	enteroinvasive *E. coli*
EHEC	enterohemorrhagic *E. coli*
EAEC	enteroaggregative *E. coli*
EPEC	enteropathogenic *E. coli*
ENS	enteric nervous system
4-EOTC	4-epi-oxytetracycline
FODMAP	fermentable oligosaccharides, disaccharides, monosaccharides, and polyols
FMT	fecal microbiota transplantation
GTT	gut transit time
IBS	irritable bowel syndrome
IBS-C	constipation-predominant IBS
IBS-D	diarrhea-predominant IBS
IBD	inflammatory bowel disease
*L. acidophilus*	*Lactobacillus acidophilus*
*L. bulgaricus*	*Lactobacillus bulgaricus*
*L. casei*	*Lactobacillus casei*
*L. paracasei*	*Lactobacillus paracasei*
*L. reuteri*	*Lactobacillus reuteri*
*L. rhamnosus*	*Lactobacillus rhamnosus*
*L. GG*	*Lactobacillus rhamnosus GG*
OTC	oxytetracycline
RCT	randomized controlled trial
RNA	ribonucleic acid
rRNA	ribosomal RNA
SIBO	small intestinal bacterial overgrowth
SCFA	short-chain fatty acid SCFA
*S. aureus*	*Staphylococcus aureus*
*S. epidemidis*	*Staphylococcus epidermidis*
*S. boulardii*	*Saccharomyces boulardii*
*S. typhi*	*Salmonella typhi*
*S. thermophilus*	*Streptococcus thermophilus*
sIgA	immunoglobulin A
TD	traveler’s diarrhea
*V. cholerae*	*Vibrio cholerae*
